# SLEEP AND CAREGIVER BURDEN FOR CAREGIVERS OF PERSONS WITH DEMENTIA: A SCOPING REVIEW

**DOI:** 10.1093/geroni/igac059.2917

**Published:** 2022-12-20

**Authors:** Meghan Mattos, Veronica Bernacchi, Kelly Shaffer, Virginia Gallagher, Shinae Seo, Carol Manning

**Affiliations:** University of Virginia, School of Nursing, Charlottesville, Virginia, United States; Penn State College of Medicine, State College, Pennsylvania, United States; University of Virginia, Charlottesville, Virginia, United States; University of Virginia, Charlottesville, Virginia, United States; University of Virginia, School of Nursing, Charlottesville, Virginia, United States; UVA, Charlottesville, Virginia, United States

## Abstract

Caregivers of persons with dementia report worse sleep when compared to the general population. This scoping review synthesizes evidence regarding the link between caregiver burden and dementia caregivers’ sleep. A systematic search was completed in PubMed, CINAHL, PsycINFO, and Web of Science for pertinent literature published through March 2022. Included original research articles studied informal adult caregivers of persons living with mild cognitive impairment or dementia, documented the relationship between subjective caregiver burden and caregiver sleep, and were written in English. Extracted data were organized in tables, compared, and synthesized. The search yielded 540 non-duplicate articles screened by title and abstract; 118 full-text articles were reviewed; of these, 26 were included. Most studies were cross-sectional, with variable sample sizes (range n=40–669). Sleep was operationalized across the 25 quantitative studies in terms of subjective quality (n=19) and objective sleep parameters using actigraphy (n=4), and polysomnography (n=2). Of studies reporting subjective sleep quality, 16 reported a significant positive association between caregiver burden (84%) with the remaining found null results. Half of the studies that used objective measures of sleep (actigraphy and polysomnography) found a positive association between sleep and caregiver burden, while the other half did not (n=3). Results suggest that, while subjective sleep quality is commonly impacted by dementia caregiving burden, there is a lack of corresponding evidence on the relationship between burden and objective sleep metrics. Caregiver burden was also not measured consistently across studies, and future studies should focus on consistent measurement of caregiver burden and determination of directionality.

